# Performance Study on Preparation of Mine Backfill Materials Using Industrial Solid Waste in Combination with Construction Waste

**DOI:** 10.3390/ma18153716

**Published:** 2025-08-07

**Authors:** Yang Cai, Qiumei Liu, Fufei Wu, Shuangkuai Dong, Qiuyue Zhang, Jing Wang, Pengfei Luo, Xin Yang

**Affiliations:** 1 School of Materlals and Architectural Engineering, Guizhou Normal University, Guiyang 550025, China; 232200191883@gznu.edu.cn (Y.C.);; 2 School of Civil Engineering, Guizhou Institute of Technology, Guiyang 550025, China

**Keywords:** ecological restoration, recycled aggregate, resource utilization, constitutive model, composite-activated cementitious material

## Abstract

The resource utilization of construction waste and industrial solid waste is a crucial aspect in promoting global urbanization and sustainable development. This study focuses on the preparation of mine backfill materials using construction waste in combination with various industrial solid wastes—ground granulated blast furnace slag (GGBFS), fly ash (FA), silica fume (SF), phosphorus slag (PS), fly ash–phosphorus slag–phosphogypsum composite (FA-PS-PG), and fly ash–phosphorus slag–β-phosphogypsum composite (FA-PS-βPG)—under different substitution rates (50%, 55%, 60%) as control parameters. A total of 19 mix proportions were investigated, evaluating their slump, dry density, compressive strength, uniaxial compressive stress–strain relationship, micromorphology, and phase composition. The results indicate that, compared to backfill materials prepared with pure cement, the incorporation of industrial solid wastes improves the fluidity of the backfill materials. At 56 days, the constitutive model parameter *a* increased to varying degrees, while parameter *b* decreased, indicating enhanced ductility. The compressive strength was consistently higher with PS at all substitution rates. The FA-PS-PG mixture with a 50% substitution rate achieved the highest 56-day compressive strength of 8.02 MPa. These findings can facilitate the application of construction waste and industrial solid waste in mine backfilling projects, delivering economic, environmental, and resource-related benefits.

## 1. Introduction

Driven by multiple factors, including population growth, economic prosperity, and urbanization, the generation of construction and demolition waste (CDW) and industrial solid waste has shown a sharp upward trend in recent years [1]. The annual global generation of CDW exceeds 3 billion tons [2]. According to data from the Guizhou Statistical Yearbook, Guizhou Province produced 47.46 million tons of CDW and 128.10 million tons of industrial solid waste in 2023. Due to lagging resource utilization technologies and low comprehensive utilization rates, the primary disposal method for CDW and industrial solid waste remains stockpiling, which has caused severe pollution to local soil, air, and water bodies [3]. Given the enormous quantity and potential utilization value of CDW and industrial solid waste, improving their high-value resource utilization rate is one of the key pathways to achieving sustainable urban development [4].

The technology for producing carbon-negative building materials from multi-source solid waste was recognized as a global engineering development frontier in the civil, hydraulic, and architectural engineering fields in 2023. Numerous researchers have investigated the synergistic utilization of CDW and industrial solid waste [5,6]. Qiusong Chen et al. [7] comprehensively evaluated the feasibility of using phosphogypsum and CDW as aggregates for cemented paste backfill through slump tests, setting time measurements, unconfined compressive strength analysis, and microstructural characterization. Static leaching tests were further conducted to assess their environmental impact on underground ecosystems. The results demonstrated that both the engineering performance and environmental indicators of the cemented paste backfill met all required standards. Yang Xiaobing and Wei Hanbo et al. [8,9] developed composite-activated cementitious materials incorporating various industrial solid wastes including ground granulated blast furnace slag (GGBFS), fly ash (FA), steel slag (SS), magnesium slag (MS), and flue gas desulfurization gypsum for backfill applications. This approach significantly reduced filling costs while enabling environmentally sustainable mining practices. Zhang Songhe et al. [10] compared different constitutive models for fly ash–carbide slag–slag recycled concrete and found that the Guo Zhenhai model achieved superior fitting performance, with correlation coefficients (R^2^) of 0.99 for both the ascending (parameter *a*) and descending (parameter *b*) curve parameters. Research has identified that construction waste exhibits characteristics such as low strength, high porosity, lightweight properties, and significant variability [11]. These unique properties make it particularly suitable for applications including mine backfilling, pervious concrete, and vegetation-growing concrete, suggesting broad potential for sustainable utilization.

The backfill mining method is one of the three traditional underground mining techniques. According to statistics, backfilling costs account for one-fifth of total mining expenses, with cementitious materials alone contributing up to three-fifths of the total backfill cost [12]. Therefore, there is an urgent practical need to develop low-cost cementitious materials for producing economical backfill materials. Shulong Liu et al. [13] synthesized alkali-activated backfill cementitious materials by employing calcium carbide residue and NaAlO_2_; as composite activators for FA and phosphorus slag (PS). Tekin Yilmaz et al. [14] investigated the compressive strength development, durability characteristics, and microstructural evolution of cemented paste backfill over 360 days using CDW as a partial replacement for tailings. Yifan Ji et al. [15] developed microbial-cemented recycled aggregate backfill through microbially induced carbonate precipitation (MICP) technology as an alternative to conventional cement-based binders. Talha Sarici et al. [16] examined the feasibility of utilizing alkali activators and GGBFS-modified CDW for backfill material production. Faeze Sadat Khandani et al. [17] systematically evaluated the physical and mechanical properties of backfill materials prepared from CDW with cement incorporation.

The utilization of CDW in combination with industrial solid wastes for mine backfill material preparation faces several technical challenges: Firstly, CDW pretreatment proves particularly difficult due to its significantly non-uniform particle size distribution and high water absorption characteristics [18,19]. Secondly, substantial variations in reactive components exist among different types of industrial solid wastes [8], both of which adversely affect the stability of material performance. Notably, current research exhibits distinct limitations in solid waste recycling applications. Most studies have employed only single or dual industrial solid wastes combined with CDW, lacking comprehensive investigation into the stress–strain curve relationships of backfill materials.

To the best of the authors’ knowledge, this study presents the first comprehensive comparative investigation on mine backfill materials prepared with varying dosages of multiple industrial solid wastes synergistically combined with CDW. Notably, six composite industrial waste formulations were systematically examined. Using CDW from Guanshanhu District, Guiyang City, as aggregate, we substituted cement at different replacement ratios (50%, 55%, 60%) with various industrial solid wastes abundantly stockpiled in Guizhou Province—including GGBFS, FA, silica fume (SF), PS, and their composites (FA-PS-PG, FA-PS-βPG). The low-cost backfill materials were prepared through proportional incorporation of water and superplasticizers. The feasibility and optimal mix design were evaluated through slump tests, dry density measurements, and compressive strength analysis. A constitutive model was established for mine backfill materials incorporating industrial solid wastes with CDW. Furthermore, scanning electron microscopy (SEM) and phase composition analysis were employed to elucidate the mechanistic effects of waste type and dosage on material performance. This research advances solid waste valorization by simultaneously addressing cost reduction and natural resource conservation, ultimately facilitating the achievement of environmental sustainability, resource efficiency, and economic benefits.

## 2. Materials and Methods

### 2.1. Experimental Materials

This study primarily utilized construction waste from Guanshanhu District, Guiyang City, as aggregates, with industrial solid wastes including GGBFS, FA, SF, PS, and PG partially replacing cement as binding materials for mine backfill preparation. The main chemical components of the construction waste were SiO_2_ and CaO [14]. X-ray fluorescence (XRF) spectroscopy was employed to analyze the chemical composition of cement and industrial solid wastes, with the results presented in Table 1.

Aggregates: The aggregates were sourced from construction waste (CDW) collected by Guiyang Hongji Building Materials Co., Ltd. in Guanshanhu District, Guiyang City, Guizhou Province, China. The waste primarily consisted of discarded concrete, mortar, bricks, and impurities such as glass, wood chips, steel, and plastics, with concrete, mortar, and bricks accounting for approximately 90% of the waste. The construction waste was crushed using a jaw crusher (Produced by Hengchang Mining Equipment Manufacturing Co., Ltd., Jiangxi Province, China) and sieved for later use. Based on crushing characteristics, performance requirements of backfill materials, and practical engineering needs, this study selected four particle size ranges (0–2.36 mm, 2.36–4.75 mm, 4.75–9.5 mm, and 9.5–16 mm) with incorporation ratios of 22.5%, 22.5%, 30%, and 25%, respectively.

Cement: The 42.5 composite cement produced by the Conch Cement Plant in Qingzhen City, Guizhou Province, China, was used. It had a fineness of 2.3% (sieve analysis method), an initial setting time of 210 min, and a final setting time of 500 min. Its chemical composition is listed in Table 1.

Ground Granulated Blast Furnace Slag (GGBFS): The GGBFS is a gray S95-grade powder purchased from Henan Yixiang New Materials Co., Ltd. in Henan Province, China, with a specific surface area of 380 m^2^/kg. Its chemical composition is presented in Table 1.

Fly Ash (FA): The fly ash was sourced from a power plant in Guizhou Province, China, with a specific surface area of 360 m^2^/kg. Its chemical composition is detailed in Table 1.

Silica Fume (SF): The silica fume was procured from Henan Yixiang New Materials Co., Ltd., Henan Province, China as a grayish-white powder, with a loss on ignition of 3.42%, a specific surface area of 400 m^2^/kg, and a chloride ion content of 0.07%. Its chemical composition is listed in Table 1.

Phosphorus Slag Powder (PS): The phosphorus slag powder was ground from slag obtained from a yellow phosphorus plant in Guizhou Province, China. It appeared as a grayish-white powder, and its chemical composition is provided in Table 1.

Phosphogypsum (PG):The phosphogypsum was obtained from Wengfu Phosphoric Acid Plant in Guizhou Province, China, appearing as a grayish-white material with a pH of 4.3. By heating to 150–180 °C, the dihydrate gypsum partially lost its crystalline water, converting into β-type hemihydrate gypsum.

Superplasticizer (SP): To enhance the workability of the backfill material, a liquid polycarboxylate-based superplasticizer produced by Guizhou stone Doctor Technology Co., Ltd. in Guizhou Province, China was selected, with a 45% solid content and 25% water-reducing rate.

### 2.2. Experimental Procedures

This study was designed with target parameters of 150–160 mm slump, 2.5–3MPa compressive strength, and cost reduction. The reference mix ratio was set as cementitious material: construction waste/water/superplasticizer = 1:5:1.55:0.02. Using construction waste as an aggregate, the research employed GGBFS, FA, SF, PS, FA-PS-PG, and FA-PS-βPG to replace cement at substitution rates of 50%, 55%, and 60% of the cementitious material. The detailed mix proportions are specified in Table 2.

**Figure 1:**
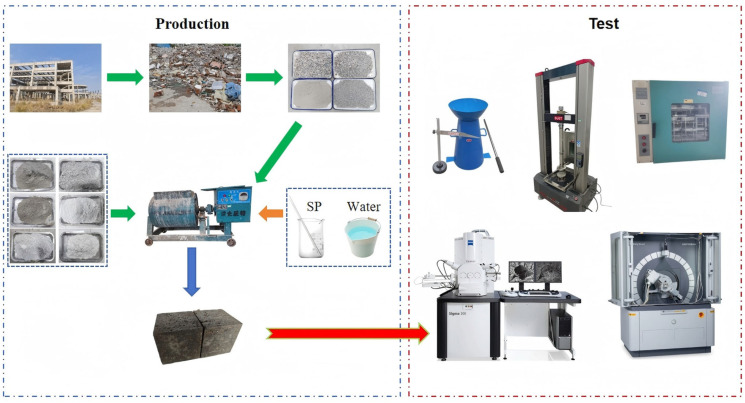
The flow chart of research. The colored arrows are used to indicate progressive logical relationships in the process flow, while the colored dashed lines group equipment/materials of the same category for easier identification.

During specimen preparation, fine aggregates, cement, and industrial solid wastes were first mixed in a mixer for 1 min. While continuing to mix, the water reducer and half of the mixing water were gradually added. Subsequently, coarse aggregates were introduced, followed by the remaining water, with mixing continuing for an additional 1 min. The slump of the thoroughly mixed concrete was then tested. The fresh mixture was cast into 100 mm × 100 mm × 100 mm molds and compacted by vibration. After 24 h of indoor curing, the specimens were demolded and placed in a standard curing room with a temperature of 20 ± 2 °C and humidity of 95 ± 2%, where they continued to cure until reaching ages of 7 days, 28 days, and 56 days. At each age, the specimens were removed to test their mechanical properties, dry density, microscopic morphology, and phase composition. The research flowchart is illustrated in Figure 1.

### 2.3. Experimental Methods

Workability: The slump test evaluated the workability of freshly mixed backfill material. First, the steel plate and the inner wall of the slump cone placed on the plate were moistened. Then the backfill material was filled into the cone in three layers, with each layer compacted by rodding. After leveling the top surface, the cone was quickly lifted vertically. The height difference after the slump stopped was measured to determine the slump value, thereby assessing the workability of the backfill material.

Dry Density: Three 100 mm cubic specimens were selected from each group of backfill materials for dry density measurement. At 28 days of age, the specimens were removed from the curing chamber and placed in a 105 °C oven until reaching constant mass, after which their oven-dried mass was measured.

Compressive Strength: The compressive strength was primarily tested using a WANCE universal testing machine (Produced by Shenzhen Wance Testing Equipment Co., Ltd., Guangdong Province, China). For each group, nine specimens needed to be prepared to measure the compressive strength at three curing ages—7 days, 28 days, and 56 days—with three specimens for each age. The average value of the test results was taken. According to the Standard for Test Methods of Concrete Mechanical Properties (GB/T 50081-2019) [20], the testing range was 0–100 kN, conducted at a displacement rate of 5 mm/min, with data collected at 0.02 s intervals until the test concluded.

Microstructure: The micromorphology of backfill material samples was observed using the Zeiss GeminiSEM 300 field emission scanning electron microscope (The equipment was manufactured by Carl Zeiss Group, Germany). Prior to testing, flake samples smaller than 1 cm×1 cm were collected and immersed in absolute ethanol to terminate hydration. The samples were then dried in an oven at 55 °C, followed by polishing and gold sputtering treatment [21].

Hydration products: The phase composition of the backfill material samples was analyzed using the Bruker D8 Advance X-ray diffractometer (The equipment was manufactured by Bruker Corporation, Germany). Prior to testing, the samples were ground into fine powder. The XRD analysis was performed with a scanning range of 5–90° (2θ) and a step size of 0.02° [22].

## 3. Results and Discussion

### 3.1. Workability

**Figure 2:**
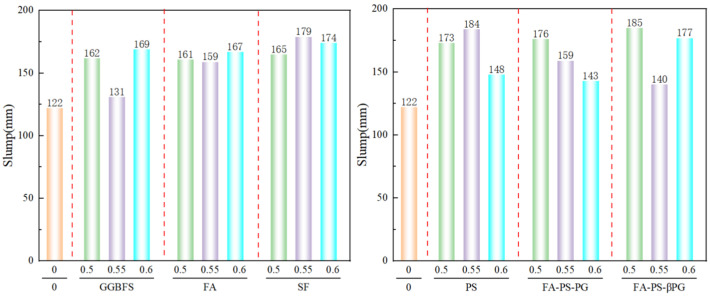
The influence of industrial solid waste types and dosages on slump. The colored bars in the figure represent the control group and different replacement ratios, while the colored dashed lines serve to distinguish between each type of industrial solid waste for enhanced visual clarity.

The materials used for mine backfilling require pumping, which imposes certain requirements on slump. The slump test results of backfill mixtures with different mix proportions are shown in Figure 2. As can be seen from Figure 2, the slump of the pure cement group (control group) was 122 mm, while the incorporation of industrial solid waste significantly increased the slump, with most improvements ranging between 30.33% and 51.64%. This indicates that the addition of industrial solid waste effectively enhances the flowability of the material, likely due to its physical characteristics (such as particle morphology) and chemical activity.

It is noteworthy that the slump increase rates of GGBFS-55%, PS-60%, FA-PS-PG-60%, and FA-PS-βPG-60% were relatively low, at 7.38%, 21.31%, 17.21%, and 14.75%, respectively. This discrepancy may be attributed to the following factors: The high dosage of GGBFS (55%) may increase the viscosity of the paste, partially offsetting the improvement in fluidity. The incorporation of PS did enhance slump, but the increase was limited, likely due to its angular, polyhedral microstructure [13]. In the FA-PS-PG and FA-PS-βPG composite systems, although the spherical particles of FA (fly ash) contributed to fluidity, the combined effects of PS and PG (or βPG) may have restricted further slump improvement.

The incorporation of FA and SF in the mixtures significantly improved the slump, which is consistent with the bearing effect of spherical SiO_2_ mentioned in reference [19]. The spherical particles of FA reduced the friction between particles, while the microfilling effect of SF optimized the particle gradation of the paste, playing a water-reducing role and thereby greatly improving the fluidity.

### 3.2. Dry Density

**Figure 3:**
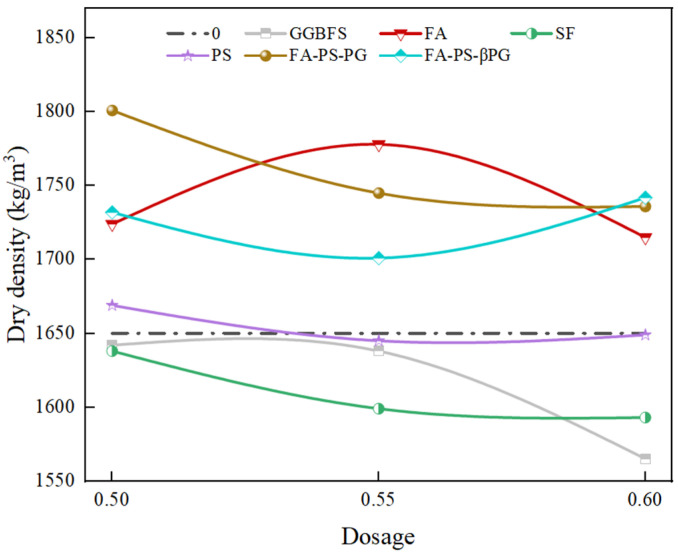
The influence of industrial solid waste on the dry density of backfill materials.

Dry density is a key indicator for evaluating the compactness of backfill materials, directly affecting their mechanical properties and long-term stability. As shown in Figure 3, the dry density of the pure cement group (control group) was 1650 kg/m^3^, while the incorporation of different industrial solid wastes resulted in dry densities ranging from 1565 to 1801 kg/m^3^. The addition of GGBFS and SF led to lower dry densities compared to the control group, and the density decreased with increasing content. Specifically, when the content of GGBFS and SF increased from 50% to 60%, the dry density decreased from 1642 kg/m^3^ to 1565 kg/m^3^ and from 1638 kg/m^3^ to 1593 kg/m^3^, respectively. This may be due to the formation of foil-like calcium silicate hydrate, which has a loose structure and large pores, resulting in reduced overall compactness [23]. The dry density of the PS-incorporated samples was similar to that of the control group, possibly because the higher chemical reactivity of the PS promoted the formation of hydration products that filled voids and cracks, leading to a denser and more stable structure.

The incorporation of FA initially increased and then decreased the dry density, indicating that its optimal replacement ratio is likely around 55%. The spherical particles of FA can optimize particle gradation and reduce pores, thereby improving compactness. However, excessive FA content may lead to the accumulation of unreacted particles, which in turn reduces density. The dry densities of the FA-PS-PG and FA-PS-βPG composite systems were significantly higher than those of the control group. This is attributed to the synergistic optimization of the microstructure by the micro-aggregate effect of FA and the activation effect of PG (or βPG), resulting in the formation of denser hydration products. The retarding characteristic of PG may prolong hydration time and promote a more uniform pore distribution, leading to the FA-PS-PG composite system achieving the highest dry density among all groups at a 50% replacement rate [7].

### 3.3. Compressive Strength

**Figure 4:**
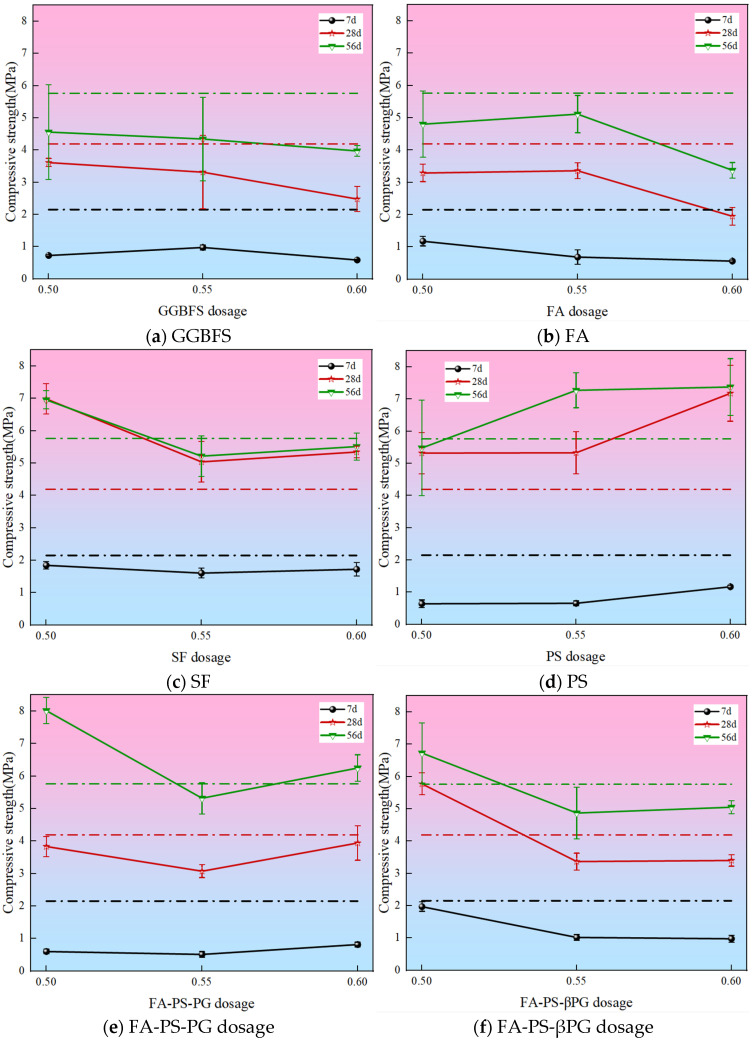
The influence of types and dosages of industrial solid waste on compressive strength. The dashed lines represent the compressive strength parameters of the control group: the black dashed line indicates the 7-day compressive strength, the red dashed line represents the 28-day compressive strength, and the green dashed line denotes the 56-day compressive strength.

Compressive strength is the most fundamental property required for mine backfill materials. Figure 4 shows the compressive strength of backfill materials with different industrial solid wastes and replacement ratios at curing ages of 7 days, 28 days, and 56 days. The pure cement group exhibited a 7 days compressive strength of 2.15 MPa, a 28 days strength of 4.29 MPa, and a 56 days strength of 5.76 MPa. The compressive strength of the backfill materials at all curing ages decreased as the replacement ratios of GGBFS, FA, and SF increased. Specifically, compared to the control group, at the 56 days curing age, the compressive strengths of GGBFS-50%, FA-50%, and SF-50% decreased by 20.83% and 16.67%, and increased by 20.83%, respectively, while the compressive strengths of GGBFS-60%, FA-60%, and SF-60% decreased significantly by 31.08%, 41.49%, and 4.34%, respectively. When SF was incorporated at 50%, 55%, and 60%, the 7 days, 28 days, and 56 days compressive strengths were consistently higher than those of the groups with GGBFS or FA. Moreover, the 28 days compressive strength of all SF-incorporated groups exceeded that of the control group, and the 56 days compressive strength of the SF-50% group was also higher than that of the control group. However, the later-stage strength growth of the SF-incorporated groups was slower. This may be attributed to the fact that silica fume contains over 90% reactive SiO_2_, which, upon activation, forms calcium silicate hydrate (C-S-H) that primarily contributes to early strength development in cement.

Backfill materials incorporating PS, FA-PS-PG, and FA-PS-βPG exhibited higher compressive strengths. Among them, the FA-PS-PG at a 50% replacement ratio achieved the highest 56 days compressive strength, surpassing the control group by 39.24%. This is likely due to the synergistic effect of multiple ions in promoting cementitious reaction efficiency, as well as the SO_4_^2−^ provided by phosphogypsum reacting with SiO_2_ and Al_2_O_3_ in PS and FA to form abundant ettringite (AFt), significantly enhancing strength—a finding supported by microstructural analysis [13,24]. The incorporation of phosphorous slag demonstrated excellent performance at all replacement ratios and curing ages, possibly because the latent hydraulic activity of phosphorous slag enables it to participate in reactions under alkaline conditions, supplementing C-S-H gel and enhancing the compactness of the interfacial transition zone.

### 3.4. Constitutive Model

**Figure 5:**
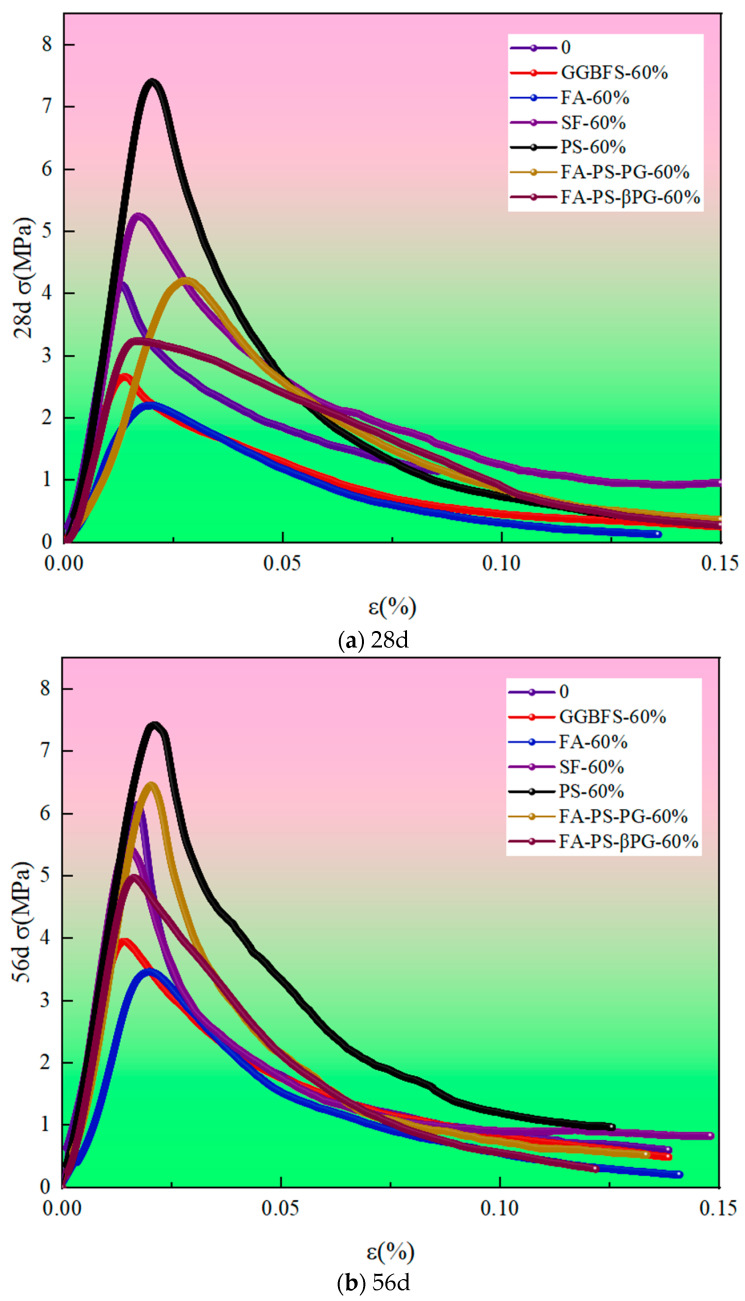
Uniaxial compression stress–strain curves of backfill materials.

As shown in Figure 5, the uniaxial compressive stress–strain curve of concrete includes characteristic indicators such as peak stress, peak strain, and ultimate strain, and studying it holds practical engineering value. Analysis of the complete uniaxial compressive stress–strain curve test of backfill materials prepared from industrial solid waste synergized with construction waste reveals that the failure mode is primarily shear failure, similar to that of ordinary concrete [25].

Considering the geometric characteristics of the stress–strain curve of the pit backfill materials prepared from industrial solid waste and construction waste, a constitutive model for these materials was established based on the classic concrete constitutive model proposed by Professor Zhenhai Guo [26]. The uniaxial compressive stress–strain curve of the backfill material was normalized, with σ/σ_m_ as the abscissa and ε/ε_m_ as the ordinate. The curve was divided into ascending and descending segments, which were fitted separately. The resulting model parameters are listed in Table 3. Here, ε_m_ and σ_m_ represent the peak strain and peak stress, respectively, while *a* is the control parameter for the ascending segment and *b* is the control parameter for the descending segment of the curve.

$$\begin{array}{cc} y & =ax+(3-2a)x^{2}+(a-2)x^{3},\;0\leq x\leq 1\\ y & =\frac{x}{{\left[b(x-1\right)}^{2}+x]},\;x>1 \end{array}$$

The control parameter *a* in the ascending curve segment represents the ratio of the initial tangent modulus to the peak secant modulus of concrete under uniaxial compression. A higher value of *a* indicates smaller early-stage deformation of the concrete but accelerated deformation in later stages. With the incorporation of industrial solid waste, the *a* values at both 28 days and 56 days increase to varying degrees. The control parameter *b* in the descending curve segment reflects the brittle characteristics of concrete. A larger *b* value corresponds to lower residual strength after peak stress, indicating greater material brittleness. After adding industrial solid waste, the *b* values at 28 days generally increase, suggesting enhanced brittleness, while the *b* values at 56 days decrease, indicating improved ductility [10]. The fitting results for both the ascending and descending segments are excellent, with R^2^ values all above 0.90 and most even reaching as high as 0.99.

### 3.5. Microstructure of Backfill Material

**Figure 6:**
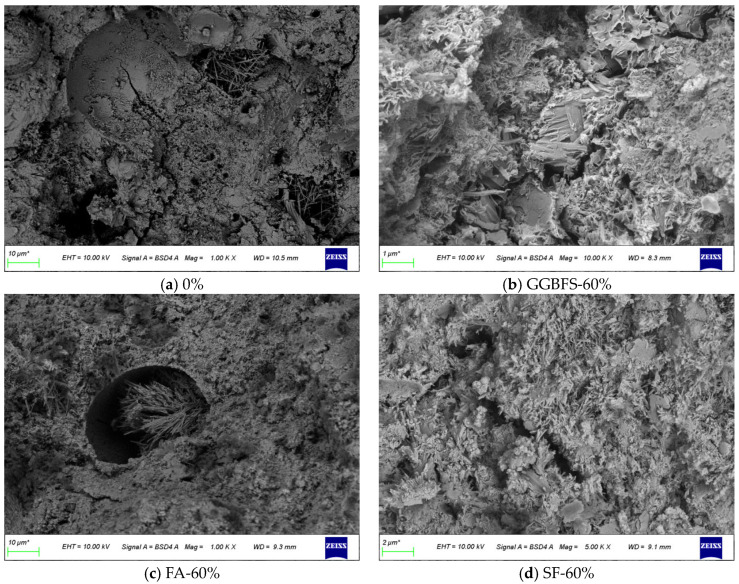

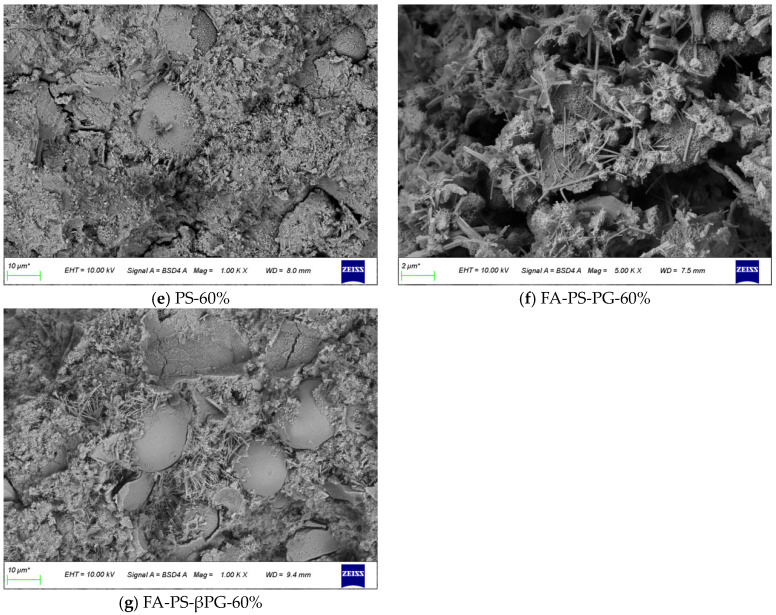
SEM images of 56d backfill materials.

Figure 6 shows the SEM images of hydration products at the matrix interface of backfill materials after 56 days of curing for the following groups: 0% (control), GGBFS-60%, FA-60%, SF-60%, PS-60%, FA-PS-PG-60%, and FA-PS-βPG-60%. The pure cement group formed dense cement stone, with existing cracks and voids filled by needle–rod-shaped AFt, creating a high-strength structural system. The incorporation of GGBFS resulted in the formation of loose and porous foil-like C-S-H [23], leading to a more porous and sunken structure with increased voids, which negatively affects compressive strength. For the FA group, a large amount of needle–rod-shaped AFt and flocculent C-S-H were generated. The aligned growth of needle–rod-shaped AFt partially filled the voids, which may be the reason for the strength reduction. The incorporation of SF reveals numerous unhydrated silica fume particles enveloped by dendritic C-S-H, with minor amounts of platy Ca(OH)_2_ being observable. In the PS group, the surface of the fly ash was being eroded and participating in hydration reactions. A significant amount of hydration products formed at the cracks, gradually filling the gaps and thereby enhancing strength. The FA-PS-PG-60% specimen exhibited abundant hydration products, including AFt, Ca(OH)_2_, and C-S-H, which interlocked to form a dense structure. In the FA-PS-βPG-60% specimen, a large amount of AFt and C-S-H filled the gaps between fly ash and aggregates. However, the fly ash had not yet participated in hydration and mainly served as a filler, leading to a slight reduction in strength [13].

### 3.6. Hydration Products of Backfill Materials

**Figure 7:**
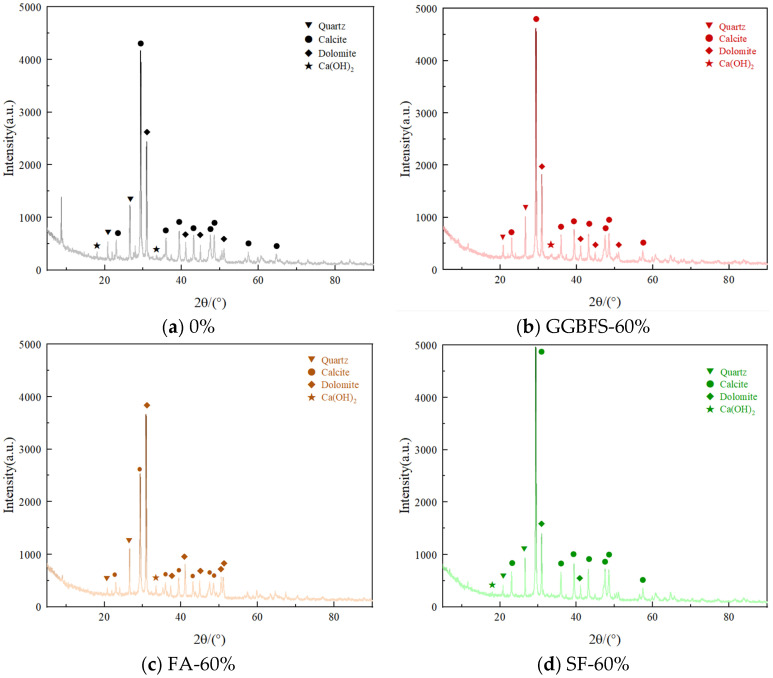

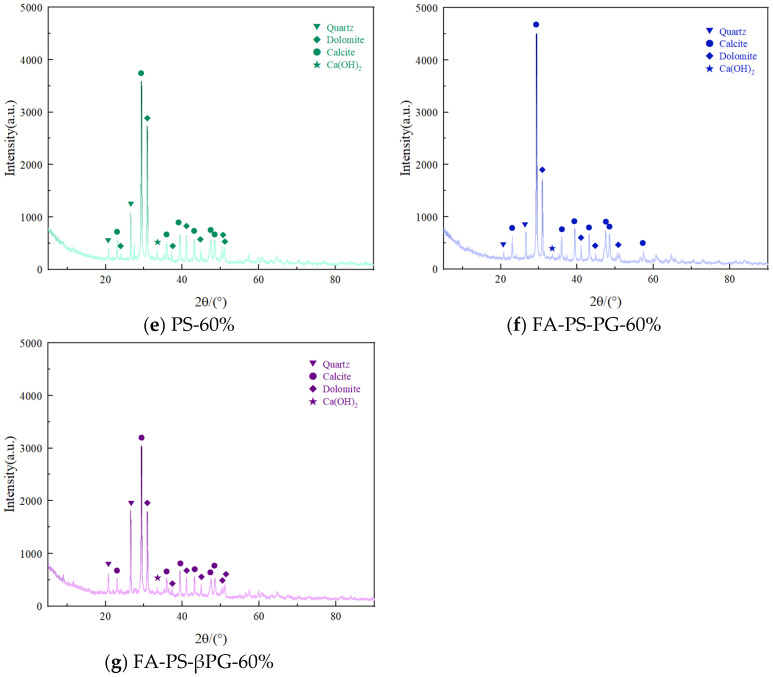
XRD results for 56d backfill materials.

Figure 7 shows the XRD analysis results for the backfill materials after 56 days of curing for the 0%, GGBFS-60%, FA-60%, SF-60%, PS-60%, FA-PS-PG-60%, and FA-PS-βPG-60% samples. As illustrated, all samples exhibit strong diffraction peaks at 26.56°, 29.34°, and 30.9°, which through phase analysis are identified as silicon dioxide, calcium carbonate, and dolomite. The CaCO_3_ primarily originates from carbonation during the curing process and nonhydratable particles in construction waste, with its formation promoting hydration reactions [27]. Notably, the FA-60% sample shows a significant reduction in the calcium carbonate diffraction peak. Only weak diffraction peaks of calcium hydroxide are observed at 18° and 33.48° in all samples, providing the necessary alkaline environment for ongoing hydration reactions. This alkalinity facilitates the pozzolanic reaction, thereby promoting the formation of C-S-H gel [28]. These findings are consistent with the microstructural characteristics observed in SEM analysis.

## 4. Conclusions

This study demonstrates the feasibility of using construction waste as an aggregate and partially replacing cement with GGBFS, FA, SF, PS, FA-PS-PG, and FA-PS-βPG to prepare backfill materials. The demonstration is based on macro-performance parameters such as slump, dry density, compressive strength, and constitutive models. Additionally, a detailed analysis of the microscopic morphology and phase composition was conducted. The raw materials used in this study were primarily sourced from Guizhou Province, and the findings are currently only applicable to Guizhou Province. Based on the research findings, the following key conclusions can be drawn:

(1) The incorporation of industrial solid waste increased the slump of backfill materials by 7.38–51.64%. The minimum slump was observed when GGBFS was added at 55%, while the maximum slump occurred with FA-PS-βPG at a 50% dosage. This enhanced fluidity facilitates the preparation of backfill materials.
(2) The incorporation of industrial solid wastes resulted in the dry density of the backfill materials ranging from 1565 to 1801 kg/m^3^. The dry density exhibited a decreasing trend with increasing dosages of GGBFS, SF, PS, and FA-PS-PG. However, the addition of FA alone led to an initial increase followed by a decrease in dry density. In contrast, the incorporation of FA-PS-βPG caused the dry density to first decrease and then increase.
(3) Comparative analysis of the compressive strength of backfill materials with different mix proportions revealed the following: Specimens incorporating PS exhibited higher compressive strength across all replacement ratios. Mixtures containing SF showed negligible strength development at later ages. The lowest 56-days compressive strength (3.37 MPa) was observed at a 60% FA replacement level. The highest 56-days compressive strength (8.02 MPa) was achieved with FA-PS-PG at a 50% replacement rate.
(4) Compared with the control group, the incorporation of industrial solid wastes resulted in varying degrees of increase in parameter a and decrease in parameter b of the 56-days constitutive model, indicating enhanced elasticity. All mixtures demonstrated good fitting performance.

This study can be applied to mine backfilling engineering, improving the utilization rate of construction waste and industrial solid waste, thereby contributing to sustainable development. Future research should focus on more in-depth investigations into the long-distance pumping performance requirements, longer-term strength development, and long-term durability of construction waste and industrial solid waste as mine backfill materials. Such efforts will help promote green and safe development in the mining industry.

## Figures and Tables

**Table 1:** Chemical composition of cement, GGBFS, PS, FS, and SF(%).

Name	SiO_2_	Fe_2_O_3_	Al_2_O_3_	CaO	MgO	SO_3_	TiO_2_	Na_2_O	K_2_O	Others
Cement	15.62	5.69	3.63	68.54	2.23	2.26	0	0.40	0.43	1.20
GGBFS	39.36	1.26	7.75	45.70	3.86	0	1.07	0	0	1.00
FA	48.32	7.97	24.78	7.75	0.82	0.55	0	0.54	3.79	5.48
SF	92.30	1.56	1.78	1.71	0.89	0	0	0.53	0	1.23
PS	38.17	1.72	3.48	44.13	1.87	0	0	0	0	10.63

**Table 2:** Mix design of different proportions and types of industrial solid waste (kg/m^3^).

Mix Code	Cement	Industrial Solid Waste						CDW	Water	SP
		GGBS	FA	SF	PS	PG	βPG			
0	234							1170	363	4.68
GGBFS-50%	115	115						1150	357	4.6
GGBFS-55%	104	127						1156	358	4.62
GGBFS-60%	92	138						1150	357	4.6
FA-50%	115		115					1150	357	4.6
FA-55%	104		127					1156	358	4.62
FA-60%	92		138					1150	357	4.6
SF-50%	115			115				1150	357	4.6
SF-55%	104			127				1156	358	4.62
SF-60%	92			138				1150	357	4.6
PS-50%	115				115			1150	357	4.6
PS-55%	104				127			1156	358	4.62
PS-60%	92				138			1150	357	4.6
FA-PS-PG-50%	115	54.25	54.25			7		1150	357	4.6
FA-PS-PG-55%	104	59.96	59.96			7		1156	358	4.62
FA-PS-PG-60%	92	65.09	65.09			8		1150	356	4.6
FA-PS-βPG-50%	115	54.25	54.25				7	1150	357	4.6
FA-PS-βPG-55%	104	59.96	59.96				7	1156	358	4.62
FA-PS-βPG-60%	92	65.09	65.09				8	1150	356	4.6

**Table 3:** Backfill material constitutive parameters *a* and *b*.

Mix Code	Ascent Stage		Descent Stage	
	*a*	R^2^	*b*	R^2^
28d-0	−0.14	0.99	0.85	0.9
28d-GGBFS-60%	0.53	0.99	0.63	0.94
28d-FA-60%	0.8	0.99	1.03	0.99
28d-SF-60%	−0.11	0.99	0.8	0.99
28d-PS-60%	−0.01	0.99	2.15	0.99
28d-FA-PS-PG-60%	−0.03	0.99	1.74	0.99
28d-FA-PS-βPG-60%	0.44	0.99	0.27	0.99
56d-0	−0.03	0.98	2.84	0.93
56d-GGBFS-60%	0.55	0.99	0.69	0.99
56d-FA-60%	−0.04	0.99	1.23	0.99
56d-SF-60%	0.37	0.99	1.58	0.98
56d-PS-60%	0.57	0.99	1.78	0.97
56d-FA-PS-PG-60%	0.02	0.99	2.65	0.98
56d-FA-PS-βPG-60%	0.25	0.99	0.91	0.99

## Data Availability

The original contributions presented in this study are included in the article. Further inquiries can be directed to the corresponding authors.
